# Disruption of Transverse-Tubules Eliminates the Slow Force Response to Stretch in Isolated Rat Trabeculae

**DOI:** 10.3389/fphys.2020.00193

**Published:** 2020-03-06

**Authors:** Amelia Power, Sarbjot Kaur, Cameron Dyer, Marie-Louise Ward

**Affiliations:** Department of Physiology, Faculty of Medical and Health Sciences, University of Auckland, New Zealand

**Keywords:** slow force response, t-tubules, ventricular trabeculae, calcium, β-adrenergic activation

## Abstract

Ventricular muscle has a biphasic response to stretch. There is an immediate increase in force that coincides with the stretch which is followed by a second phase that takes several minutes for force to develop to a new steady state. The initial increase in force is due to changes in myofilament properties, whereas the second, slower component of the stretch response (known as the “slow force response” or SFR) is accompanied by a steady increase in Ca^2+^ transient amplitude. Evidence shows stretch-dependent Ca^2+^ influx during the SFR occurs through some mechanism that is continuously active for several minutes following stretch. Many of the candidate ion channels are located primarily in the t-tubules, which are consequently lost in heart disease. Our aim, therefore, was to investigate the impact of t-tubule loss on the SFR in non-failing cardiac trabeculae in which expression of the different Ca^2+^ handling proteins was not altered by any disease process. For comparison, we also investigated the effect of formamide detubulation of trabeculae on β-adrenergic activation (1 μM isoproterenol), since this is another key regulator of cardiac force. Measurement of intracellular calcium ([Ca^2+^]_i_) and isometric stress were made in RV trabeculae from rat hearts before, during and after formamide treatment (1.5 M for 5 min), which on washout seals the surface sarcolemmal t-tubule openings. Results showed detubulation slowed the time course of Ca^2+^ transients and twitch force, with time-to-peak, maximum rate-of-rise, and relaxation prolonged in trabeculae at optimal length (L_o_). Formamide treatment also prevented development of the SFR following a step change in length from 90 to 100% L_o_, and blunted the response to β-adrenergic activation (1 μM isoproterenol).

## Introduction

Ventricular myocytes are the “working” cells of the heart, responsible for force generation. Their structure is characterized by regular invaginations of the surface sarcolemma (SL) that occur at the Z disc. These invaginations form a complex tubular system that is continuous with the extracellular environment, commonly referred to as the “transverse tubules” (or “t-tubules”). Linkages between t-tubules are also present in the longitudinal axis of myocytes (Sperelakis and Rubio, 1971), providing a highly branched tubular complex. The t-tubules have an important role during excitation-contraction (EC) coupling, ensuring that the action potential is propagated rapidly, and evenly, throughout the myocytes, which is essential for synchronized contraction. During the cardiac action potential, influx of Ca^2+^ through voltage-gated, L-type, Ca^2+^ channels triggers Ca^2+^ release into the cytosol by activating ryanodine receptors (RyR) in the junctional membrane of the sarcoplasmic reticulum (SR), which is the myocyte intracellular Ca^2+^ store. This causes a ∼10-fold increase in cytosolic [Ca^2+^]_i_, during the “Ca^2+^ transient,” which initiates cross-bridge cycling and force production. Many of the ion channels essential to EC coupling and Ca^2+^ transport are present in greater density in the t-tubule SL, which makes up 64% of the total sarcolemmal area of rat ventricular myocytes (Soeller and Cannell, 1999). These include the L-type Ca^2+^ channels which are in close apposition to the RyR clustered at the junctional SR (Forbes and Sperelakis, 1983). This specialized region, known as the “dyad,” is critical to simultaneous Ca^2+^-induced Ca^2+^-release throughout the myocyte.

Relaxation of cardiac myocytes is brought about by the cytosolic [Ca^2+^]_i_ returning to resting (diastolic) levels. Although the main protein responsible for removal of cytosolic Ca^2+^ is the sarco-endoplasmic reticulum ATPase (SERCA), the Na^+^-Ca^2+^ exchanger (NCX) also contributes. NCXs are also concentrated in the myocyte t-tubule SL (Thomas et al., 2003), and are important in Ca^2+^ removal from the junctional region of the dyad where Ca^2+^ reaches very high concentrations during RyR release. NCX is electrogenic, operating in “forward mode” to extrude 1 Ca^2+^ ion in exchange for 3 Na^+^ ions (Hinata et al., 2002; Bers and Ginsburg, 2007), as well as in reverse mode to bring additional Ca^2+^ into the myocytes at positive membrane potentials (Litwin et al., 1996).

Disruption and/or loss of myocyte t-tubule structure has been reported in myocytes from human (Crossman et al., 2011) and many animal models (Wei et al., 2010; Ward and Crossman, 2014; Power et al., 2018) of heart disease in which contraction is compromised. The resulting increased t-tubule heterogeneity has been suggested as causing prolonged action potential duration (Beuckelmann et al., 1993; Tomaselli and Marban, 1999), asynchrony of subcellular Ca^2+^ release, and reduced force in diseased myocytes (Heinzel et al., 2011). However, the question remains as to what contribution t-tubule loss has on reduced contraction, since many of the pathologies associated with heterogenous t-tubules also exhibit alterations in Ca^2+^ cycling and myofilament proteins. This makes it difficult to assign the contractile dysfunction to t-tubule disruption, *per se*.

Therefore, our aim was (i) to examine the impact of acutely disrupting t-tubules on [Ca^2+^]_i_ and force in *multicellular* ventricular trabeculae, and (ii) to investigate the impact of detubulation on the slow force response to stretch which relies predominantly on transport of ions across the t-tubule SL. We also investigated the effects of detubulation on the β-adrenergic response which, along with stretch, is a key regulator of cardiac contraction. We hypothesized that pharmacological detubulation of trabeculae from healthy hearts would have an impact on both the magnitude of the SFR and on the response to β-adrenergic stimulation since both mechanisms augment force by receptor activation and/or ion transport across the t-tubule SL. However, β-adrenergic receptors are distributed throughout both the surface and t-tubule sarcolemma (Cros and Brette, 2013), therefore we anticipated the functional response to β-adrenergic stimulation would only be partially blunted by detubulation.

## Materials and Methods

### Ethical Approval

This study was carried out in strict accordance with the recommendations in the Guide for the Care and Use of Laboratory Animals of the National Institutes of Health. The experimental protocol was approved by the Animal Ethics Committee of The University of Auckland (permit number AEC 001232). Animals were anaesthetized with isoflurane using 100% O_2_ as the carrier gas until reflexive responses were lost and then killed by decapitation. Hearts were removed and perfusion of the coronary circulation with oxygenated solution (see below) maintained throughout dissection.

### Isolation, Dissection, and Mounting of Trabeculae

Un-branched trabeculae (cross-sectional area: 0.057 ± 0.010 mm^2^) were micro-dissected from the right ventricle (RV) of adult Wistar rats (weight: 287 ± 6 g, *n* = 13) with small blocks of ventricular wall at each end. Trabeculae were transferred to a bath (450 μl) on the stage of an inverted microscope (Nikon Eclipse TE2000-U, Japan) and continuously superfused with oxygenated physiological solution (see below). The small block of tissue at one end of the trabecula was held in a wire cradle extending from the beam of a force transducer (KX801 Micro Force Sensor, Kronex Technologies), while the tissue block at the other end was held in a nylon snare attached to a manipulator used to impose length changes. Field stimulation was provided at 0.2 Hz by electrical pulses of 5 ms duration at a voltage 10% above threshold (model D100, Digitimer^TM^, United Kingdom). Once trabeculae were contracting at steady state, loading with fura-2/AM was commenced.

### Measurement of [Ca^2+^]_i_

Intracellular calcium ([Ca^2+^]_i_) measurements were made as described previously (Ward et al., 2003). Briefly, trabeculae were loaded for 2 h at room temperature (21.0 ± 0.1°C) with 10 μM fura-2/AM (Teflabs, TX, United States) dissolved in 30 μl of anhydrous DMSO with 5% wt/vol of Pluronic F-127 (Teflabs, TX, United States). Fluorescence was measured at 510 nm for excitation wavelengths of 340, 360, and 380 nm. The 340/380 ratio of emitted fluorescence was obtained as a measure of [Ca^2+^]_i_. Emitted fluorescence at 360 nm did not vary with stimulation, or formamide treatment, confirming there was no movement artifact.

### Experimental Interventions

Formamide exposure was carried out in trabeculae after baseline control data was obtained for each experimental intervention. 1.5 M formamide was added to the superfusate for 5 min whilst continually monitoring [Ca^2+^]_i_ and isometric force. Two experimental protocols were carried out that were known to have a high dependence on membrane channels and transporters predominantly located in the t-tubule SL.

#### The Slow Force Response to Stretch

Investigation of the slow force response to stretch was carried out before and 5 min after wash out of formamide. Trabeculae were first shortened to 90% of their optimal length (L_o_) and allowed to reach steady state before rapidly lengthening to 100% L_o_ where they were held for 3 mins before decreasing again to 90% L_o_. The Ca^2+^ transient and twitch force parameters immediately following the stretch were then compared with those after 3 min at 100% L_o_.

#### ß-Adrenergic Activation

The response to ß-adrenergic activation was also investigated. One micrometer isoproterenol, a non-selective β adrenoreceptor agonist (Martin and Broadley, 1994), was added to the superfusate before (control) and after formamide treatment in trabeculae, with a return to isoproterenol-free steady state after each intervention.

### Confocal Imaging of Trabeculae

Confocal imaging of trabeculae was carried out to confirm detubulation following formamide treatment by staining trabeculae with wheat germ agglutinin (WGA) and ryanodine receptor (RyR) antibodies. Control and formamide treated trabeculae (1.5 M formamide for 5 min followed by 10 min wash out) were held at L_o_ and fixed in 2% PFA for 10 min at room temperature. Trabeculae were then incubated for 2 h with Image-iT FX signal enhancer (Invitrogen Life Technologies, United States) followed by washing in phosphate buffer solution (PBS) 3 × 5 min. Next, trabeculae were immunolabeled with RyR monoclonal primary antibody (C3-33, catalog # MA3-916, Invitrogen Life Technologies, United States) (1:100, diluted in 1% bovine serum albumin with 0.1% Triton X-100 in PBS) followed by overnight incubation at 4°C. Next day, trabeculae were washed in PBS 3 × 5 min followed by 3 h incubation with goat anti-mouse Alexa Fluor 594 (catalog # A-11005, Invitrogen Life Technologies, United States) (1:200) and WGA conjugated Alexa Fluor 488 (1:200) at room temperature. Trabeculae were then washed in PBS, the tissue blocks at either end removed, and mounted on a slide with Prolong Gold antifade reagent (Invitrogen Life Technologies, United States). Confocal imaging of trabeculae was carried out to confirm detubulation in formamide treated trabeculae in comparison to control by viewing for t-tubular striations from WGA and RyR stains using 63 × objective with oil under Olympus FV1000 confocal microscope.

### Chemicals and Solutions

Trabeculae were continuously superfused with a HCO_3_^–^-free Tyrode’s solution bubbled with 100% O_2_, containing (in mM): 141.8 NaCl; 6 KCl; 1.2 MgSO_4_; 1.2 Na_2_HPO_4_; 10 HEPES; 10 D-Glucose adjusted to pH 7.4. Extracellular calcium ([Ca^2+^]_o_) was set by adding CaCl_2_ from a 1 M stock solution. The dissection and initial mounting solution contained 0.25 mM [Ca^2+^]_o_, and 20 mM 2,3-butanedione monoxime (BDM) for cardio-protection (Mulieri et al., 1989). Once mounted in the muscle chamber, trabeculae were superfused with a BDM-free solution. [Ca^2+^]_o_ was raised to 1.0 mM during fura-2 loading, and then to 1.5 mM during experiments (unless otherwise indicated). All chemicals were purchased from Sigma (Sigma Aldrich, Australia) unless stated otherwise.

### Data Analysis

Data were acquired using LabVIEW (National Instruments, Austin, TX, United States) and analyzed off-line using a custom-written program (IDL Research Systems Inc., Boulder, CO, United States). Ca^2+^ transients were fitted by a five parameter function as described previously (Ward et al., 2003). All results were expressed as mean ± SEM. Statistical analysis was performed using paired or unpaired Student’s *t*-tests, as well as two-way analysis of variance (ANOVA) followed by Sidak’s multiple comparisons test using Prism 7.0c (GraphPad software, CA, United States) depending on the experimental protocol. A statistically significant difference was assumed at *P* < 0.05.

## Results

**FIGURE 1:**
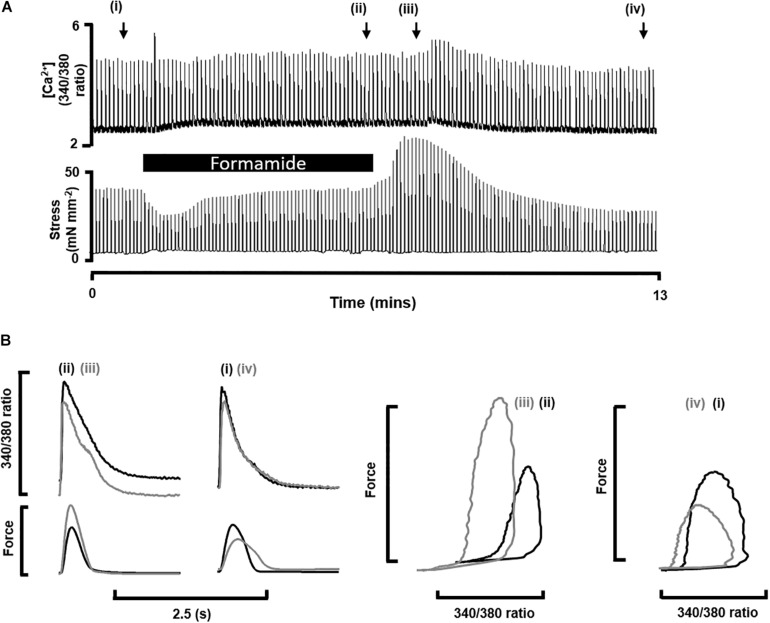
Impact of formamide on multicellular trabeculae. **(A)** Shows continuous trace of [Ca^2+^]_i_ as fura-2 340/380 fluorescence (top) and isometric force (bottom) recorded from a representative trabecula stimulated at 0.2 Hz before (i), during (black bar, ii) and after (iii,iv) exposure to 1.5 M formamide. The x-axis shows time, and the black bar indicates 5 min of formamide exposure. **(B)** Shows examples of individual [Ca^2+^]_i_ and twitch force traces from the time points labeled in **(A)**.

To demonstrate the effects of formamide on [Ca^2+^]_i_ and force throughout the whole detubulation process we show a continuous record of simultaneous Ca^2+^ transients (fura-2 340/380 fluorescence) and their corresponding twitch force from a representative trabecula before, during and after exposure to 1.5 M formamide. Figure 1A shows diastolic and peak [Ca^2+^]_i_ was increased during formamide exposure (ii), returning to pre-formamide control levels after ∼5 min of formamide washout (iv). Peak force also increased during formamide, with no discernible change in the diastolic force. Comparison of Ca^2+^ transient and twitch force kinetics before and after formamide treatment are shown in Figure 1B. Region (iv) showed lower amplitudes of both Ca^2+^ transients and twitch force in comparison to pre-formamide control (i), with a ∼20 ms delay in reaching peak force post-formamide treatment, as is illustrated by the gray lines on the force-time plot which are superimposed in time for comparison.

Table 1 shows mean ± SEM data from n = 9 trabeculae for the time points labeled in Figure 1A. The time to peak [Ca^2+^]_i_ was longer during and after formamide treatment, with the time constant of Ca^2+^ transient decay prolonged during the formamide exposure (ii), but not different to the pre-formamide control after washout. Active stress was decreased during and after formamide, with slower relaxation, although the maximum rate-of-rise of stress increased. An additional line (v) was added to Table 1 for *n* = 4 trabeculae showing data 30 min after formamide washout. Although the Ca^2+^ transients in these trabeculae were not different to pre-formamide controls, the delay in time to peak [Ca^2+^]_i_ and time to 50% relaxation of force continued, suggesting the surface SL t-tubule openings remained sealed for this period of time.

**TABLE 1:** Mean ± SEM [Ca^2+^]_i_ transient (340/380 fura-2 ratio) and twitch force for the time points shown in Figure 1: before (i), during (ii), immediately after (iii), 5 min after (iv), and 30 min after (v) formamide treatment.

Time point	n	Diastolic [Ca^2+^]_i_(340/380 ratio)	Systolic [Ca^2+^]_i_(340/380 ratio)	Time to peak [Ca^2+^]_i_(s)	Tau [Ca^2+^]_i_ decay(s)	Max rate of rise in stress(mN mm^–2^ s^–1^)	Active stress(mN mm^–2^)	Time to 50% relaxation(s)	Peak [Ca^2+^]_i_ to peak force delay (s)
(i)	9	2.03 ± 0.13	3.60 ± 0.25	0.074 ± 0.004	0.24 ± 0.02	0.027 ± 0.002	40.3 ± 10.5	0.18 ± 0.01	0.145 ± 0.009
(ii)	9	2.18 ± 0.14*	3.94 ± 0.22**	0.092 ± 0.004**	0.40 ± 0.03**	0.043 ± 0.009**	28.1 ± 4.5**	0.14 ± 0.01*	0.102 ± 0.008***
(iii)	9	1.96 ± 0.14*	3.90 ± 0.31**	0.087 ± 0.003*	0.31 ± 0.04*	0.055 ± 0.012**	36.9 ± 5.8*	0.15 ± 0.01**	0.104 ± 0.007***
(iv)	9	2.06 ± 0.14	3.45 ± 0.23*	0.083 ± 0.005**	0.24 ± 0.02	0.032 ± 0.012**	31.6 ± 8.4*	0.22 ± 0.02**	0.159 ± 0.014
(v)	4	1.82 ± 0.07	2.78 ± 0.16	0.084 ± 0.003*	0.22 ± 0.01 (*P* = 0.06)	0.019 ± 0.004**	14.9 ± 5.1	0.20 ± 0.02**	0.144 ± 0.013*

Data are from n = 9 trabeculae for time points (i) to (iv). Data n = 4 trabeculae are shown for time point (v). *P < 0.05, **P < 0.01, and ***P < 0.001. Two-tailed paired t-test in comparison to pre-formamide control (i).

### Slow Force Response to Stretch

**FIGURE 2:**
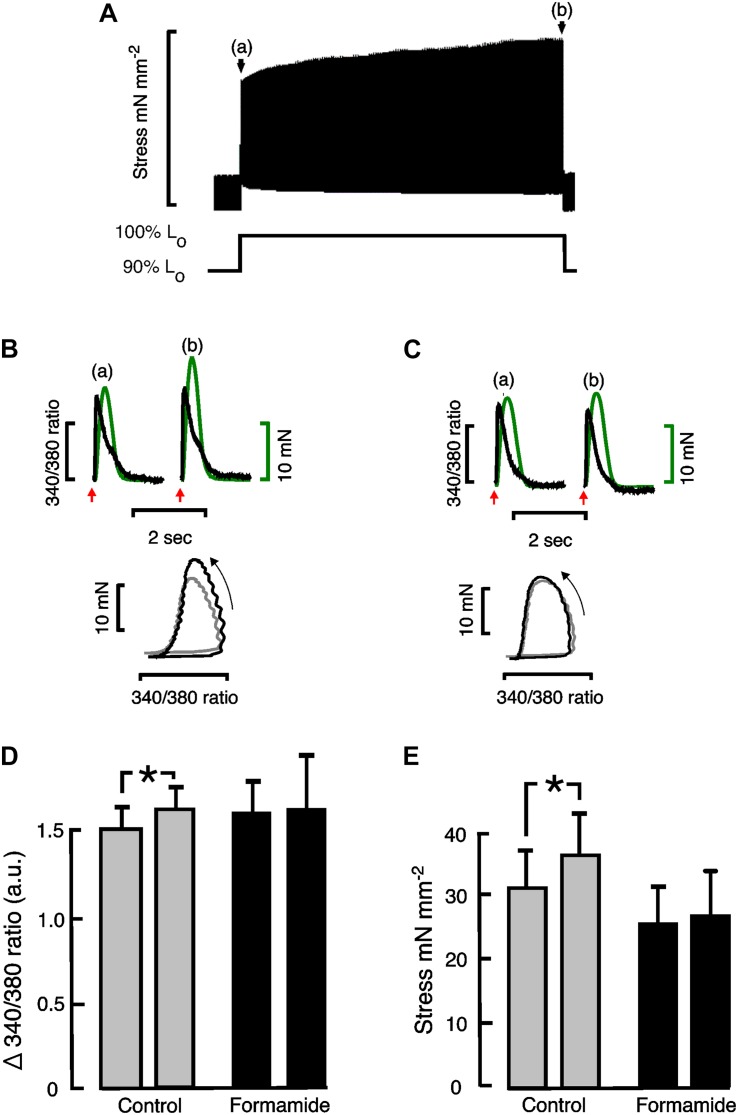
Effect of formamide on slow force response to stretch. **(A)** Shows a representative slow force response from a rat trabecula stretched from 90 to 100% L_o_. **(B)** (Control) and **(C)** (formamide) show Ca^2+^ transients (black) and twitch force (green) immediately after stretching a representative trabecula from 90 to 100% L_o_ on left (a), and after 3 min at 100% L_o_ on right (b). Red arrows indicate the time of stimulation. Phase plots of the force vs. Ca^2+^ data are shown beneath the Ca^2+^ transients and twitches with arrows indicating the direction of time. Phase plots immediately after the stretch are shown in gray, and after 3 min at 100% L_o_ are shown in black. **(D)** Shows mean ± SEM Ca^2+^ transient amplitudes (*n* = 4), and **(E)** shows mean ± SEM stress (*n* = 6) immediately after stretching trabeculae on left, and after 3 min at 100% L_o_ on right, for control (gray bars) and formamide treated (black bars) trabeculae. *P < 0.05. Two-tailed paired *t*-test. a.u, Arbitrary units.

Stretching cardiac muscle results in a biphasic increase in the force of contraction. There is an immediate increase in the force of contraction that coincides with the stretch, which is followed by a slower developing force increase that takes several minutes to reach a maximum, known as the “slow force response” or SFR. The SFR is accompanied by increasing Ca^2+^ transients that result from stretch-dependent Ca^2+^ influx via transport proteins thought to be located predominantly in the t-tubules. We therefore investigated whether detubulation would eliminate the SFR. Following trabeculae stretch there was an immediate increase in active stress, which was not affected by formamide treatment (Control: 148 ± 37% vs. Formamide: 129 ± 32%; *P* = 0.45, *n* = 5 trabeculae) suggesting detubulation had no impact on the Frank-Starling mechanism. Active stress increased in Control trabeculae stretched from 90 to 100% L_o_ showing a SFR after 3 min of 114% (from 32.5 ± 5.7 mN mm^–2^ to 37.0 ± 6.9 mN mm^–2^, *n* = 6, *P* < 0.05). An example SFR from a control trabecula is shown in Figure 2A.

Figure 2B shows calcium transients with their corresponding twitches superimposed and their phase plots below from a representative control trabecula immediately following the stretch (a) and after 3 min of stretch (b). Similar Ca^2+^ transients, twitches and phase plots from the same representative trabecula are shown after formamide treatment in Figure 2C. The relaxation part of the force-calcium phase plots provides an indication of myofilament Ca^2+^ sensitivity in intact trabeculae since the crossbridge cycling is in equilibrium at this point. Figures 2B,C show similar slopes for the relaxation section of the phase plots, providing further evidence that the SFR is not associated with a change in myofilament sensitivity, unlike the immediate response to stretch (Frank-Starling mechanism).

Figure 2D shows mean Ca^2+^ transient amplitude data from control and formamide treated trabeculae. There was a small but significant increase in Ca^2+^ transient amplitude during the SFR in control trabeculae that was not observed following formamide treatment. Consequently for formamide treated trabeculae, the SFR was reduced to 104% (from 25.6 ± 6.35 mN mm^–2^ to 26.6 ± 6.7 mN mm^–2^ after 3 min of stretch, *n* = 6, NS; Figure 2E).

### Response to ß-Adrenergic Stimulation

**FIGURE 3:**
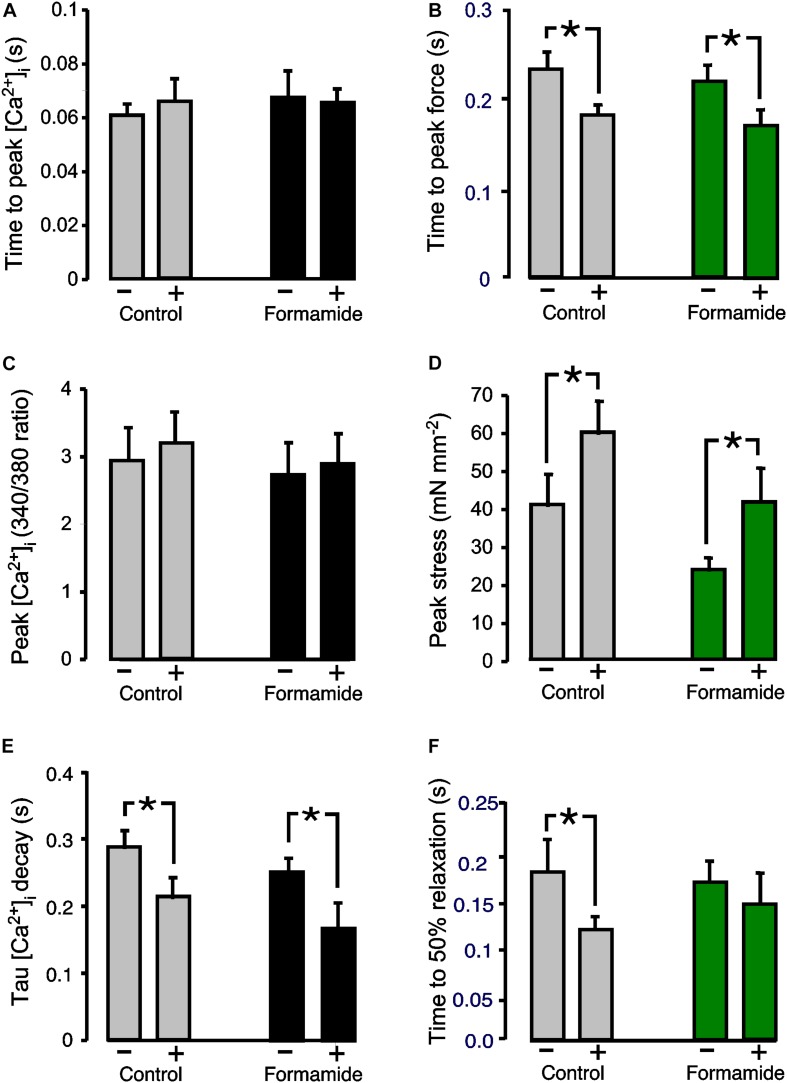
Effect of formamide on response to ß-adrenergic stimulation. **(A,C,E)** Show Ca^2+^ transient parameters before (−) and during (+) 1 μM isoproterenol. **(B,D,F)** Show twitch force parameters before (−) and during (+) isoproterenol. Data are mean ± SEM. *P < 0.05. Two-way ANOVA followed by Sidak’s multiple comparisons test (Prism), *n* = 7 control and 8 formamide-treated trabeculae.

Another important regulator of cardiac contractility is ß-adrenergic activation. Since myocyte ß-adrenergic receptors are found both on the surface, and t-tubule, sarcolemma, we hypothesized that the response to ß-adrenergic activation would be only partially depressed in formamide treated trabeculae. The ß-adrenergic effects were investigated by adding 1 μM isoproterenol to the superfusate. Figure 3 shows mean ± SEM data from *n* = 7 control and *n* = 8 formamide treated trabeculae. No effect of isoproterenol was observed for either group on the time to peak of the Ca^2+^ transients (Figure 3A), or on the amplitude of the Ca^2+^ transients (control *P* = 0.08; formamide treated *P* = 0.10; Figure 3C), but there was a decrease in the time constant of Ca^2+^ transient decay (Figure 3E) for both control and formamide treated trabeculae. Isoproterenol decreased the time to peak stress (Figure 3B), and increased peak stress (Figure 3D), by similar amounts (although from different pre-isoproterenol values) in both groups. However, the time to 50% relaxation of stress was decreased only in control but not in formamide treated trabeculae (Figure 3F).

### Evidence of Detubulation

**FIGURE 4:**
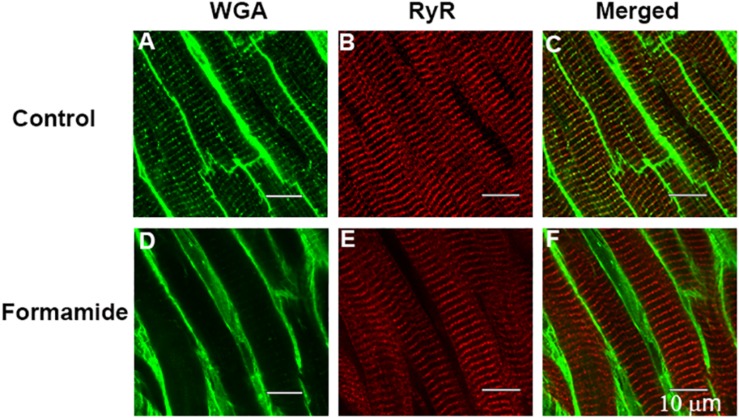
Evidence of detubulation in response to formamide. Confocal images are shown of representative ventricular trabeculae labeled with t-tubule marker WGA (green) and anti-RyR antibody (red). Top row shows **A**: WGA, **B**: RyR, and **C**: merged labelling in a control trabecula. Bottom row shows **D**: WGA, **E**: RyR, and **F**: merged labelling in a formamide treated trabecula. Scale bar 10 μm.

Confocal imaging of trabeculae dual labeled with WGA and RyR antibodies was carried out to confirm that our formamide protocol effectively sealed the SL t-tubule openings. WGA binds to sialic acid residues on the surface SL and within the t-tubules and is commonly used as a label for the extracellular matrix and SL. Images from representative trabeculae in Figure 4 show WGA labeled t-tubules in a control trabecula (Figure 4A) with an absence of t-tubule staining evident in the formamide treated preparation (Figure 4D). A similar RyR labeling pattern was observed in both control (Figure 4B) and detubulated trabeculae (Figure 4E), suggesting formamide treatment had no effect on the SR junctional ultrastructure.

## Discussion

The main goals of this project were to utilize multicellular ventricular trabeculae to (i) measure the changes in the time course of Ca^2+^ transients and isometric twitches following pharmacological removal of t-tubules; (ii) investigate the impact of detubulation on the slow force response to mechanical stretch; and (iii) determine whether detubulation modified the response to ß-adrenergic activation, since the ß1 receptors are not restricted to the t-tubule SL (Bhargava et al., 2013).

### T-Tubule Loss Effects on Multicellular Trabeculae

Formamide has been used in the past to detubulate isolated ventricular myocytes (e.g., Kawai et al., 1999; Brette et al., 2002; Despa and Bers, 2007), and to a limited extent in multicellular trabeculae (Ferrantini et al., 2014). Exposure to physiological buffer solution containing 1.5 M formamide causes myocytes to shrink and equilibrate to a new cell volume (Kawai et al., 1999). Upon formamide wash off, the resulting rapid expansion of cells causes osmotic shock which disarticulates the t-tubular openings from the surface SL (Kawai et al., 1999). In our study, formamide was added to the superfusate of multicellular trabeculae held at fixed length (100% L_o_). Simultaneous recordings of Ca^2+^ transients and isometric force were made before, during and after 5 min of formamide exposure. During formamide, we observed increased diastolic [Ca^2+^]_i_ and larger Ca^2+^ transients in trabeculae, with reduced stress. We also observed a prolonged time to Ca^2+^ transient peak and a slower time constant of decay (Table 1). This is consistent with osmotic water loss effectively increasing the cytosolic [Ca^2+^]_i_ concentration, and impairing Ca^2+^ cycling, since measurements were made using a ratiometric Ca^2+^ indicator (fura-2).

Peak systolic [Ca^2+^]_i_ was decreased following formamide washout, and the time course of both the Ca^2+^ transients and twitch force were slower. This is consistent with impaired excitation-contraction coupling which has previously been reported after detubulation of isolated myocytes due to reduced L-type Ca^2+^ current (Gadeberg et al., 2017). This would also explain the reduced force after recovery from washout.

Measurements made 30 min after formamide washout [Table 1, (v)] suggests the surface SL t-tubule openings remained sealed since the time to peak systolic [Ca^2+^]_i_, and the time to 50% relaxation of force, remained prolonged. The Ca^2+^ transients were reduced in amplitude, and peak stress very much less after 30 mins of washout in comparison to pre-formamide treatment. Confocal imaging showed WGA had not penetrated the t-tubule system in formamide treated trabeculae, confirming that the t-tubule openings had remained sealed over (Figure 4). In contrast, no change in the RyR labeling pattern in formamide treated trabeculae was observed in comparison to control, suggesting that the junctional SR remained structurally intact and in close apposition to the t-tubule SL.

### Loss of the Slow Force Response Following Detubulation

The slow force response is a physiological response to stretch that aids contractility. The magnitude of the slow force response is reduced in failing human myocardium, despite an increase in intracellular [Na^+^], suggesting that Na^+^-contraction coupling is impaired in failing myocardium (von Lewinski et al., 2009). Since NCX resides largely in the t-tubules (Gadeberg et al., 2017), remodeling of the t-tubules could explain this lack of Na^+^ mediated increase in intracellular [Ca^2+^].

A number of studies have implicated the Na^+^-H^+^ exchanger (NHE1) as contributing to Na^+^ influx during the SFR (Calaghan and White, 2004; Kockskamper et al., 2008; von Lewinski et al., 2009; Shen et al., 2013), with an increase in intracellular [Na^+^] reported during sustained stretch− interventions (Calaghan and White, 2004; Caldiz et al., 2007). Blockade of NHE1 with pharmacological agents (Caldiz et al., 2007; Shen et al., 2013), or silencing NHE1 gene expression (Perez et al., 2011) blunts the SFR substantially in a number of species. Detubulation is unlikely to prevent stretch activation of NHE1 as these exchangers are restricted to the surface SL of cardiac myocytes (Garciarena et al., 2013). However, modeling studies attribute the stretch-dependent influx of Na^+^ to be through stretch-activated, non-specific, cation channels (SAC_NSC_), rather than via NHE1. TRPC channels are mechanosensitive, and have been implicated in the Ca^2+^ and/or Na^+^ influx during the SFR (Yamaguchi et al., 2017). Antibody labeling of TRPC6 and TRPC3, likely candidates for stretch-dependent Ca^2+^ and Na^+^ influx, shows they are abundant in isolated ventricular myocytes and apparently restricted to location in the t-tubule SL (Jiang et al., 2014). Since both NCX and TRPC channels are in the t-tubule SL, we speculate that detubulation would therefore prevent any stretch-dependent increase in [Ca^2+^]_i,_ despite any possible activation of NHE1 in the surface sarcolemma.

Autocrine/paracrine factors released during stretch are also a component of the SFR and are important to consider. Others have shown that angiotensin II and endothelin are mediators of the SFR and consequently their receptors are reported to be found in the t-tubules (Chung et al., 2008). Therefore, it is plausible that their contribution to the SFR could be diminished following detubulation. However, under our experimental conditions we have found no contribution of Ang II to the SFR in rat ventricle (Shen et al., 2013). Instead, we have identified prostaglandin F2α as a stretch derived inotropic factor that contributes to the SFR (Shen et al., 2016). The distribution of F type prostaglandin receptors between the surface sarcolemma and t-tubules is unknown but could contribute to a lack of SFR in detubulated trabeculae.

### ß-Adrenergic Activation of the Detubulated Myocardium

Isoproterenol had similar effects on control and formamide treated trabeculae (Figure 3), apart from the time to 50% stress relaxation which was slower in the formamide treated trabeculae. β1-adrenergic receptors are the most abundant of the β-adrenergic receptors in ventricular myocytes. They are distributed over both t-tubule and surface SL, which perhaps explains the lack of a difference between control and formamide treated groups. β2-adrenergic receptors are restricted to the t-tubule SL in ventricular tissue (Schobesberger et al., 2017), and can produce the same effects as β1-adrenergic receptors, although they can couple to both stimulatory and inhibitory G protein coupled receptors (Triposkiadis et al., 2009). We have previously shown that loss of t-tubules in right ventricle hypertrophy coincides with an attenuated inotropic response to β-adrenergic stimulation (Power et al., 2018). However, the results from this study suggest that detubulation *per se* does not explain the impaired β-adrenergic responsiveness observed in heart failure.

## Conclusion

Our study tested the hypothesis that the slow force response to stretch requires an intact t-tubular system to enable stretch-dependent Ca^2+^ influx during the slow augmentation of force. We subjected multicellular trabeculae from healthy rat hearts to superfusion with 1.5 M formamide, which prevented establishment of the SFR. Confirmation that formamide treatment disarticulated t-tubules from the extracellular environment was obtained by WGA labeling and confocal microscopy. Our study shows for the first time that that removal of the t-tubules in healthy trabeculae impairs the response to ventricular stretch.

## Data Availability Statement

The datasets generated for this study are available on request to the corresponding author.

## Ethics Statement

This study was carried out in strict accordance with the recommendations in the Guide for the Care and Use of Laboratory Animals of the National Institutes of Health. The experimental protocol was approved by the Animal Ethics Committee of The University of Auckland (permit number AEC 001232).

## Author Contributions

M-LW contributed to the conceptualization. AP, SK, and CD contributed to the experimental investigations. M-LW, SK, AP, and CD contributed to the data analysis. M-LW, SK, and AP contributed to the manuscript preparation. M-LW contributed to the funding acquisition and administration.

## Conflict of Interest

The authors declare that the research was conducted in the absence of any commercial or financial relationships that could be construed as a potential conflict of interest.
